# Depot-Dependent Impact of Time-Restricted Feeding on Adipose Tissue Metabolism in High Fat Diet-Induced Obese Male Mice

**DOI:** 10.3390/nu15010238

**Published:** 2023-01-03

**Authors:** Theresa Bushman, Te-Yueh Lin, Xiaoli Chen

**Affiliations:** Department of Food Science and Nutrition, University of Minnesota, Twin Cities, MN 55108, USA

**Keywords:** time-restricted feeding, adipose tissue, metabolism, nutrition, obesity, high-fat diet

## Abstract

Time-restricted feeding (TRF) is known to be an effective strategy for weight loss and metabolic health. TRF’s effect on metabolism is complex and likely acts on various pathways within multiple tissues. Adipose tissue plays a key role in systemic homeostasis of glucose and lipid metabolism. Adipose tissue dysregulation has been causally associated with metabolic disorders in obesity. However, it is largely unknown how TRF impacts metabolic pathways such as lipolysis, lipogenesis, and thermogenesis within different in adipose tissue depots in obesity. To determine this, we conducted a 10-week TRF regimen in male mice, previously on a long-term high fat diet (HFD) and subjected the mice to TRF of a HFD for 10 h per day or ad libitum. The TRF regimen showed reduction in weight gain. TRF restored HFD-induced impairment of adipogenesis and increased lipid storage in white adipose tissues. TRF also showed a depot-dependent effect in lipid metabolism and restored ATP-consuming futile cycle of lipogenesis and lipolysis that is impaired by HFD within epididymal adipose tissue, but not inguinal fat depot. We demonstrate that TRF may be a beneficial option as a dietary and lifestyle intervention in lowering bodyweight and improving adipose tissue metabolism.

## 1. Introduction

The continuous increase in the numbers connected to obesity, diabetes, and their relationship, has led to a direct association in the increase in research studies focused on the effects of diets on adipose tissue metabolism, insulin resistance, energy expenditure, and the cardiovascular system. A high-fat diet (HFD) in mice has been shown to cause obesity, insulin resistance, and dyslipidemia [1]. Therefore, diets that can aid in controlling obesity can affect the impact of obesity on diabetes and hopefully reduce the upward trends of both chronic disorders. Intermittent fasting (IF) is a potential method that could play an intricate role in reducing the prevalence of diabetes and obesity [2,3]. Time-restricted feeding (TRF) is a form of IF that limits the daily time window of energy intake consumption, and it is understood that TRF can alter metabolic processes within the body. TRF has been used as a strategy for weight loss without altering caloric intake and has shown to improve blood pressure, insulin sensitivity, and oxidative stress within human studies [4,5]. This knowledge can lead to TRF’s possible association with decreasing the prevalence of diabetes and obesity.

Adipose tissue plays a critical role in the regulation of energy metabolism. A major contributor to the etiology of the above-mentioned diseases is thought to be chronic, low-grade inflammation stemming from adipose tissue. Therefore, being able to understand how TRF impacts the regulation of adipose tissue metabolism and function within an obese model is crucial and could be beneficial to addressing the continuous increase of obesity occurring in the world. The mechanism of TRF is complex and likely acts on various pathways within the adipose tissue. Currently, no TRF studies have been conducted in mice after being subjected to a long-term high fat diet (mimicking the Western diet), which provides a more accurate model of the target population. Additionally, while various studies have reported the metabolic benefits of TRF, understanding of adipose tissue depot specific response is limited. Adipose tissue is generally divided into white adipose tissue (WAT) and brown adipose tissue (BAT), differing in their metabolic functions, morphology, and gene expression. WAT, broken into two depots—subcutaneous or visceral, is the main site for energy storage and has single large lipid droplet spherical cells between 25–200 μm, whereas BAT contain multiple small lipid droplets between 15–60 μm [6], playing a role in energy expenditure through thermogenesis. Within WAT, the storage of energy is stimulated by insulin which promotes glucose and fatty acid uptake by the adipocyte and then converted to triglycerides [7]. Then, when energy levels are low, the body signals for the breakdown of stored triglycerides via the pathway of lipolysis, leading to the release of glycerol and fatty acids that can be distributed to tissues for energy use. Both WAT and BAT are also recognized as an endocrine organ which synthesizes and releases a variety of factors or so called adipokines such as leptin and adiponectin regulating whole body metabolic activities [8,9].

Adipose cell size differs based on location (WAT vs. BAT) and can change based on the amount of fat storage. During the consumption of excess calories, adipose tissue is expanded to store excess energy intake through both hyperplasia and hypertrophy mechanisms. Studies on HFD-induced adipose tissue growth have demonstrated that both adipocyte hyperplasia and hypertrophy occur during the early stage of HFD feeding [10,11]. However, as HFD feeding prolongs, the adipocyte size enlargement continues, whereas the total adipocyte numbers decline in white adipose depots [11], suggesting that long-term HFD feeding impairs adipogenesis and adipose tissue expansion in the late stage is driven mainly by hypertrophy. An increase in the fat cell size is traditionally considered to correlate with system insulin resistance and metabolic dysregulation within humans [12,13,14]. Weight loss has been shown to improve adipocyte physiology within obese individuals, with reductions in fat cell size [15]. Therefore, understanding the impact TRF has on fat cell sizing is crucial to understanding the benefits it holds as a dietary and lifestyle intervention.

The aim of this study was to investigate the impact of TRF on adipose tissue morphology and metabolism in different fat depots in high fat diet-induced obese male mice. We found that TRF can be an effective approach for preventing weight gain in HFD-induced obese male mice. TRF reverses HFD-induced metabolic changes in adipose tissues in a depot-dependent manner. The outcomes of this study help understand better the mechanism for the metabolic effect of TRF as well as learn whether TRF is a viable method to reduce obesity and its related diseases.

## 2. Materials and Methods

### 2.1. Animal Study

Animals were housed at 22 °C in a specific pathogen-free facility at the University of Minnesota. Animal studies were conducted with the approval of the University of Minnesota Animal Care and Use Committee and conformed to the National Institute of Health guidelines for laboratory animal care (IACUC 2102A38852). The study consisted of eighteen 3-month-old C57BL/6 male mice (The Jackson Laboratory, Bar Harbor, ME, USA). 14 of the mice were fed a high-fat diet (HFD) for 18 weeks to induce obesity while 4 served as the control on a normal chow diet. Following the 18 weeks, the mice were placed into one of three groups: normal chow ad libitum (Control), high-fat diet ad libitum (HFD-AL), and HFD with time-restricted feeding (HFD-TRF). The AL groups had access to food and the TRF group had access to food 10 h/day. The mice were housed in groups of 3–4 per cage, with water ad libitum and in 12 h light/dark cycles. The high-fat diet provided to the mice was a 60% HFD (Bio-Serv: F3282) and the normal chow was provided by the animal facility (Envigo: 2918). All three experimental groups were regulated by being moved between cages with and without food. All mice were moved at 8:30 PM and 6:30 AM daily for 10 weeks during the TRF portion of the study. Food intake was measured daily by cage and divided by the number of mice within the cage. After 10 weeks of the dietary intervention, all three groups were sacrificed following a 16 h fast. Blood was collected through cardiac puncture. Brown adipose tissue, inguinal adipose tissue, epididymal adipose tissue, retroperitoneal fat, kidneys, liver, and muscle were collected and weighed. Tissue section from brown adipose tissue were used for histological analysis, while small portions of inguinal and epididymal adipose tissue were used for fat cell sizing and the remaining tissue was snap-frozen via liquid nitrogen and stored for later analysis.

### 2.2. Fat Cell Sizing

Adipose tissue was obtained from inguinal and epididymal white adipose tissue of mice fed control or HFD with or without TRF. Tissue samples (25–30 mg) were immediately fixed in 12 mL of the 2% osmium tetroxide solution in collidine buffer in a Wheaton vial (SPI-Chem no. 986704) and incubated in a water bath at 37 °C for 48 h as described previously [16,17]. Collidine buffer was prepared from a 4 °C stock collidine buffer of 0.2 M 2,4,6-trimethyl-pyridine (C-0505; Sigma Chemical Co) dissolved in distilled water. Subsequently, the contents of the vial were washed out using a 25-µm filter to catch the fixed cells and the container was rinsed three more times following. After a final rinse using 0.9% saline, the material was transferred into a 250-µm filter using a squirt bottle filled with 0.9% saline. A gentle rub on the 250-µm mesh was applied to crush any large chunks and then rinsed again with 0.9% saline. The procedure was repeated to collect the cells. The end volume did not exceed more than 1 conical tube (50 mL). Samples were analyzed with a Microtrac Bluewave which is a particle characterization tool. The Bluewave is able to measure particles in the size range 50 nm to 2800 μm. Each sample was measured at least in duplicate. Figures were generated using GraphPad Prism version 9 for macOS (GraphPad Software, San Diego, CA, USA).

### 2.3. Hematoxylin and Eosin Staining of Tissues

Tissues were fixed in 10% neutral buffered formalin (VWR International, LLC, Radnor, PA, USA), then dehydrated by ethanol solutions and processed for embedding in paraffin. Tissue samples were H&E stained using a standard protocol at the University of Minnesota Histology Core. Briefly, after deparaffinization and rehydration, tissues were sectioned with 5–6 μm thickness and stained in Hematoxylin for one minute and rinsed with distilled water. After hematoxylin staining, the tissues were counterstained with Eosin solution for one minute, followed by dehydration through 95% EtOH and 100% EtOH and Xylene clearance. At last, the tissue sections were mounted with resinous mounting medium. Images were then captured using a Leica microscope.

### 2.4. Quantitative Real-Time PCR

Total RNA from frozen tissue was prepared using TRIZOL reagent (Invitro, Carlsbad, CA, USA). RNA was DNAase-treated prior to the synthesis of cDNA using Superscript II reverse transcription kit (Invitrogen, Carlsbad, CA, USA). Real-time quantitative PCR was conducted using FastStart Universal SYBR Green Master (Rox) (Roche) with a QuantStudio™ 3 Real-time PCR System (Applied Biosystem, Foster City, CA, USA). The ΔΔCt method was used to calculate mRNA expression. For quantification *Tbp* mRNA served as an endogenous control within inguinal and brown adipose tissue. β-Actin served as the endogenous control within the epididymal tissue. The primer sequences for amplifying the target genes are shown in Table S1.

### 2.5. Serum Analyses

Serum triglyceride level was determined using enzymatic assay kit (Stanbio Laboratory, Boerne, TX, USA). Serum free fatty acids and *β*-hydroxybutyrate levels were determined using free fatty acid quantification kit and *β*-hydroxybutyrate assay kit (Sigma) following manufacturer’s instructions.

### 2.6. Statistical Analysis

Results were expressed as mean ± SEM. Food intake data was measured by cage and divided by number of mice within the cage. Data was analyzed by student t-test and one-way ANOVA via GraphPad Prism (version Prism 9.4.1). *p*-values less than 0.05 were considered to be significant.

## 3. Results

### 3.1. TRF Reduces Weight Gain in HFD-Fed Male Mice

**Figure 1:**
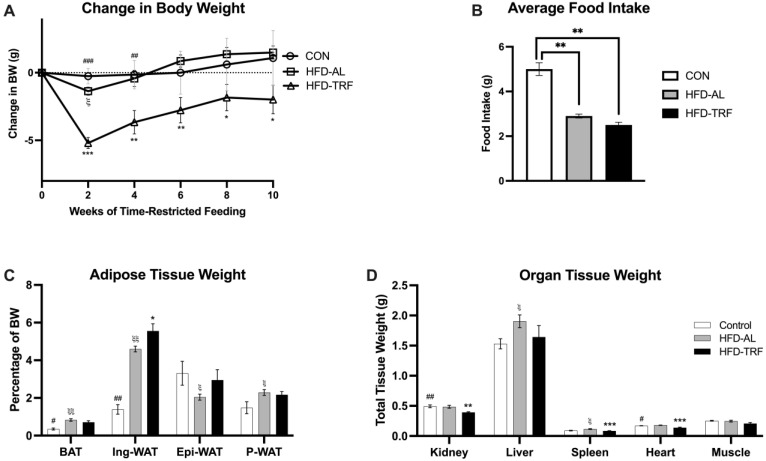
Body weight, food intake, and tissue weight of male mice. (**A**) Change in body weight during the 10 weeks of TRF from male mice on normal chow (control), high-fat diet ad libitum (HFD-AL) and high-fat diet TRF (HFD-TRF), *n* = 4–7/group. (**B**) Average food intake from male mice during the 10 weeks TRF implementation. (**C**) Adipose tissue weight from brown adipose tissue (BAT), inguinal white adipose tissue (Ing-WAT), epididymal white adipose tissue (Epi-WAT), and perirenal white adipose tissue (P-WAT). (**D**) Weights of non-adipose tissues and organs. The data are presented as mean +/− SEM. ξ: Indicates statistical significance between the control and HFD-AL. ξξ: *p* < 0.01 for Control vs. HFD. #: Indicates statistical significance between the control and HFD-TRF. ##: *p* < 0.01 for Control vs. TRF. ###: *p* < 0.001 for Control vs. TRF. *: Indicates statistical significance between the HFD-AL and HFD-TRF. **: *p* < 0.01 for HFD vs. TRF. ***: *p* < 0.001 for HFD vs. TRF.

To determine whether TRF can reverse diet-induced obesity, 12-week-old male C57BL/6 mice were placed into one of three groups following eighteen weeks of a HFD or NC diet. These three groups were: NC (control diet; 18% fat), HFD-AL (access to HFD 24/7; 60% fat), or HFD-TRF for 10-weeks (14 h fast/10 h feeding). TRF mice on HFD had significant weight loss at the beginning of the feeding regimen (Figure 1A). Throughout the 10 weeks, the TRF weight was significantly reduced compared to the HFD-AL group (Figure 1A). Overall, by the end of the study, the TRF mice were 4% lower from their starting body weight, whereas the HFD group had gained 3% body weight during that portion of the study, resulting in a 7% difference between the two groups. Food intake was measured during the study with no significant difference seen between the HFD-AL and HFD-TRF, however the TRF group was on average lower (Figure 1B). When looking at tissue weight normalized to total body weight, an increase in both brown adipose tissue (BAT) and inguinal white adipose tissue (Ing-WAT) were seen in the HFD-AL group compared to the control group (Figure 1C). The TRF group had reduced brown fat but further increased Ing-WAT with statistical significance compared to the HFD-AL group (Figure 1C). Interestingly, epididymal white adipose tissue (Epi-WAT) was reduced (*p* < 0.04) in the HFD-AL group compared to the control group; the TRF reversed this HFD-caused reduction to the level of control group (Figure 1C). The TRF group had reduced tissue weight size in BAT and P-WAT (perirenal white adipose tissue) compared to the HFD-AL group. However, TRF further increased tissue weight in inguinal and epididymal depots compared to HFD-AL group. Non-adipose tissue weight was shown to be lower in TRF mice than the HFD mice (Figure 1D). Overall, this data indicates that a TRF regimen of 14 h fast/10 h feeding is effective at preventing weight gain in mice fed HFD due to changes in non-adipose tissue weight.

### 3.2. TRF Increases the Average Fat Cell Size within the Inguinal and Epididymal Adipose Tissue of HFD-Induced Male Obese Mice

**Figure 2:**
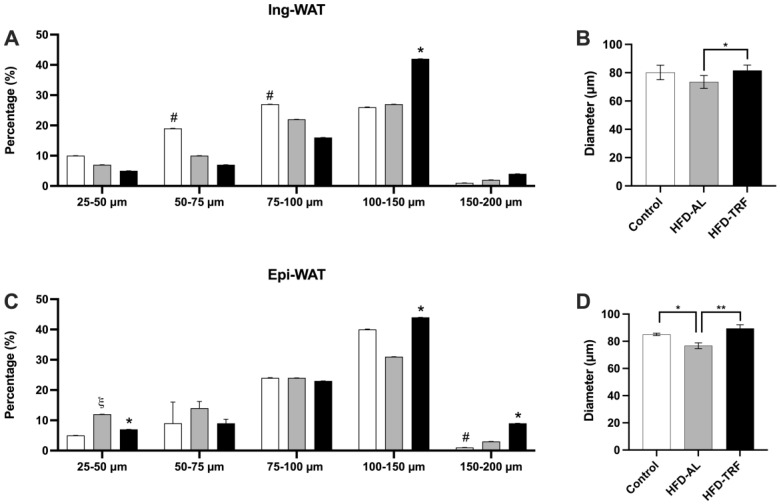
Fat cell diameter and sizing distribution within inguinal and epididymal white adipose tissue. (**A**,**B**) Size population distribution and average diameter within the inguinal white adipose tissue. (**C**,**D**) Size population distribution and average diameter within the epididymal white adipose tissue. Duplicates were run for each sample. *n* = 6–14/group. The data are presented as mean +/− SEM. ξ *p* < 0.05 for Control vs. HFD; # *p* < 0.05 for Control vs. TRF; * *p* < 0.05 for HFD vs. TRF; ** *p* < 0.01 for HFD vs. TRF.

To determine the impact of TRF on the plasticity of adipose tissue, inguinal and epididymal white adipose tissue were sectioned and fixed in an osmium tetroxide solution. Bluewave laser diffraction instrument was used to determine the differences in adipocyte size and distribution between the experimental groups. The data has shown the average size of adipose cells had a decreasing trend in the inguinal fat depot and a significant decrease in the epididymal fat depot within the HFD group compared to a normal chow diet (Figure 2B,D). This change is primarily due to increased small fat cell populations and decreased percentage of larger fat cells as shown in the cell size distribution of adipose tissue (Figure 2A,C). However, TRF mice had a significantly increased average size of adipose cells in both inguinal (*p* < 0.02) and epididymal (*p* < 0.01) fat depots compared to the HFD group (Figure 2B,D). These results suggest that TRF can reverse the HFD-induced disruption in adipogenesis and reduction in the lipid storage capacity within the inguinal and epididymal fat depots.

### 3.3. TRF Reverses HFD-Induced Whitening of Brown Adipose Tissue and Alters Mitochondrial Genes in Brown Adipose Tissue

**Figure 3:**
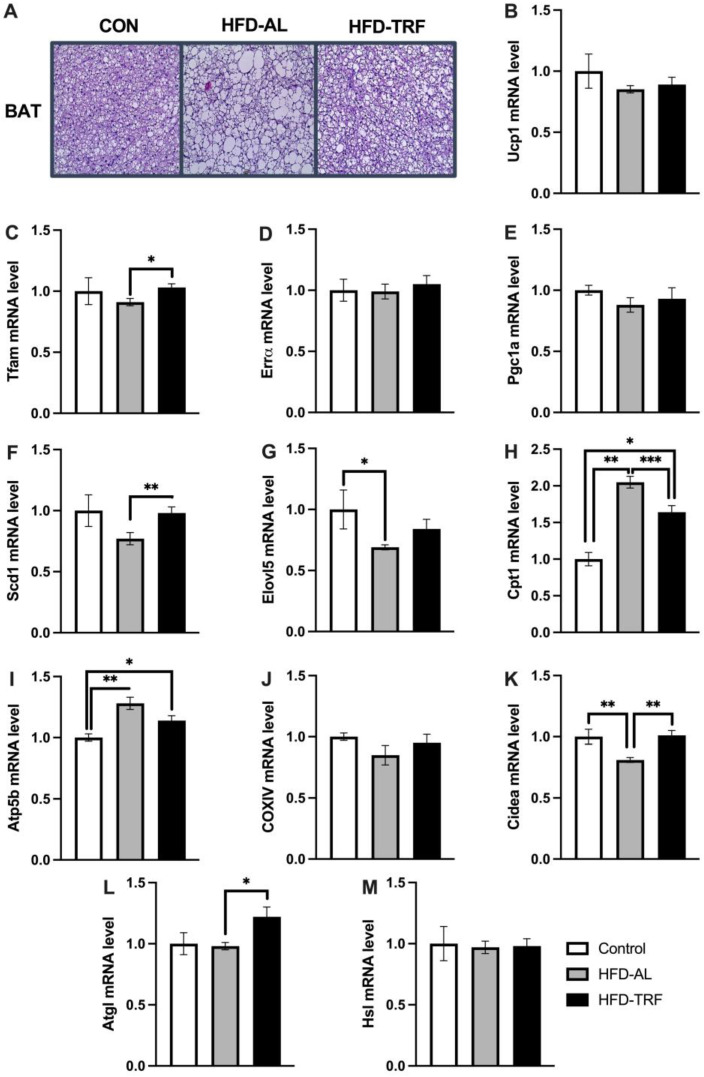
Histology of brown adipose tissue and brown adipose tissue metabolic genes. (**A**) H&E staining of brown adipose tissue between control, HFD, and TRF groups. (**B**–**M**) mRNA expression of genes involved in thermogenesis, mitochondrial biogenesis, fatty acid oxidation and lipolysis in brown adipose tissue of control, HFD-AL and HFD-TRF mice. The data are represented as mean +/− SEM. * *p* < 0.05; ** *p* < 0.01; *** *p* < 0.001.

An H&E staining of the brown adipose tissue was conducted to determine how the 10-week TRF regimen impacted the morphology of BAT. The H&E staining showed an increase in adipocyte size in BAT in the HFD-AL group compared to the control group (Figure 3A). Within the HFD-TRF mice, a reduction of adipocyte size in BAT was seen compared to the HFD-AL mice (Figure 3A).

High fat diet is known to contribute to the dysregulation of genes involved in thermogenesis and mitochondrial function within the brown adipose tissue. Therefore, qPCR analysis was done to see how the TRF regimen altered the expression of genes involved in mitochondrial biogenesis, thermogenesis, and fatty acid oxidation. Specifically, the mRNA expression of genes *Ucp1*, *Tfam, Errα*, and *Pgc-1α* were examined. As shown in Figure 3B–E, *Ucp1* expression levels has a decreasing trend in the HFD group compared to the control group, whereas TRF had no significant effect on *Ucp1* gene expression. The mRNA expression levels of *Tfam, Errα*, and *Pgc-1α* were similar between the HFD and control group. TRF trended in an increase of the expression of *Tfam, Errα*, and *Pgc-1α* levels, with significance seen for *Tfam*, a transcriptional factor that functions in maintaining mitochondrial genome (mtDNA) and regulating mitochondrial biogenesis and contents (Figure 3C) [18].

*Scd1* and *Elovl5* are the target genes of *Pparg*, playing a role in de novo lipogenesis. *Scd1* is upregulated in response to cold stimulation and involved in thermogenesis [19,20]. The mRNA expression levels of *Scd1* and *Elovl5* were decreased by HFD compared to the control group, with *Elovl5* being significantly reduced (Figure 3F,G). *Scd1* expression was significantly increased compared to the HFD group and reversed back by TRF to the level of the control group. *Elovl5* followed a similar trend. Among three genes involved in mitochondrial and fatty acid oxidation (Figure 3H–J), *Cpt1* and *Atp5b* were upregulated significantly by HFD compared to the control group (Figure 3H,I). Both genes were reduced by TRF compared to the HFD group, with *Cpt1* being significantly reduced. *Cidea* has been recently reported to play a positive role in the improvement of metabolic profile in diet-induced obesity via promoting the expansion of white adipose tissue [21]. *Cidea* gene expression was decreased significantly by HFD, which was reversed back to the control level by TRF (Figure 3K). Next, we looked at lipolytic mRNA expression levels of *Atgl* and *Hsl* genes. *Atgl* was found to be increased significantly by TRF compared to HFD, while *Hsl* had no change between the three groups (Figure 3L,M). These results indicate that TRF restores the expression of genes involved in mitochondrial biogenesis but not genes involved in *Ucp1*-dependent thermogenesis such as *Ucp1* and *Pgc-1α* in BAT. TRF also increases lipolysis (*Atgl*) and lipogenesis (*Scd1* and *Elovl5*) but reduces fatty acid oxidation in BAT.

### 3.4. TRF Restores Adipogenic and Lipolytic Gene Expression in Epididymal White Adipose Tissue

**Figure 4:**
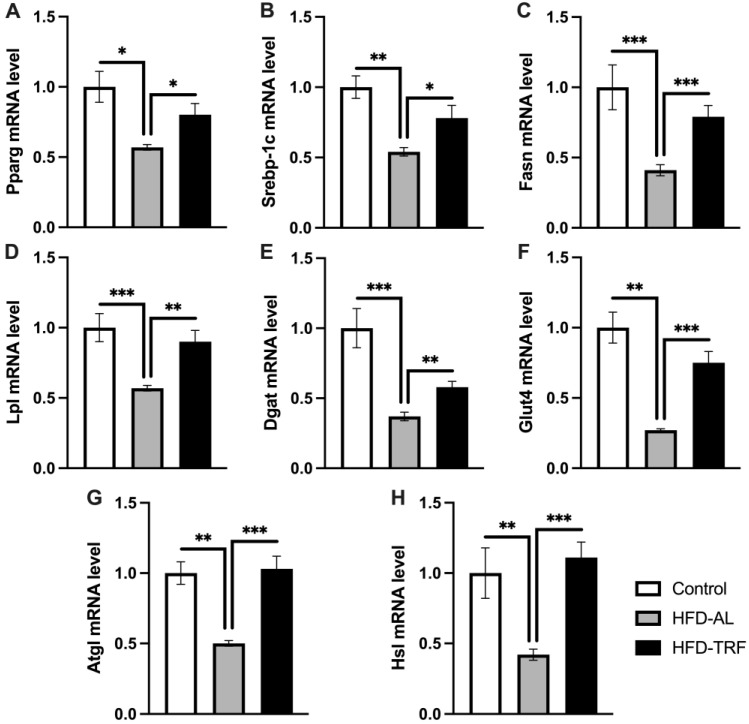
Lipolytic and adipogenic gene expression in epididymal white adipose tissue. (**A**–**H**) mRNA expression of genes involved in adipogenesis/lipogenesis and lipolysis in epididymal white adipose tissue of control, HFD-AL and HFD-TRF mice. The data are represented as mean +/− SEM. * *p* < 0.05; ** *p* < 0.01; *** *p* < 0.001.

To understand how TRF impacted white adipose tissue metabolism and function, the expression of various metabolic pathway genes within the two white adipose depots was determined. When investigating the gene expression within the epididymal tissue, adipogenic and lipolytic pathways were of interest. *Pparg* and *Srebp1c* are transcription factors, which regulate adipogenic gene expression. Both *Pparg* and *Srebp1c* genes were significantly decreased by HFD compared to the control group (Figure 4A,B). TRF mice had significantly increased mRNA expression levels for *Pparg* and *Srebp1c* compared to the HFD group, bringing the gene expression towards the control level. *Pparg* target genes involved in adipogenesis, *Fasn, Lpl, Dgat*, and *Glut4*, followed the same trend with significant decreases in expression within the HFD group compared to the control group (Figure 4C–F). Next, the lipolytic gene expressions of *Atgl* and *Hsl* were determined to provide insight into the effect of TRF while on a long-term HFD. *Atgl* and *Hsl* gene expressions were found to be decreased significantly by HFD, which was then reversed back to the control level by TRF (Figure 4G,H). Collectively, these results indicate that TRF restores the HFD-induced changes in the expression of adipogenic (lipogenic) and lipolytic genes in Epi-WAT.

### 3.5. TRF Increases Lipolytic Gene Expression within the Inguinal White Adipose Tissue

**Figure 5:**
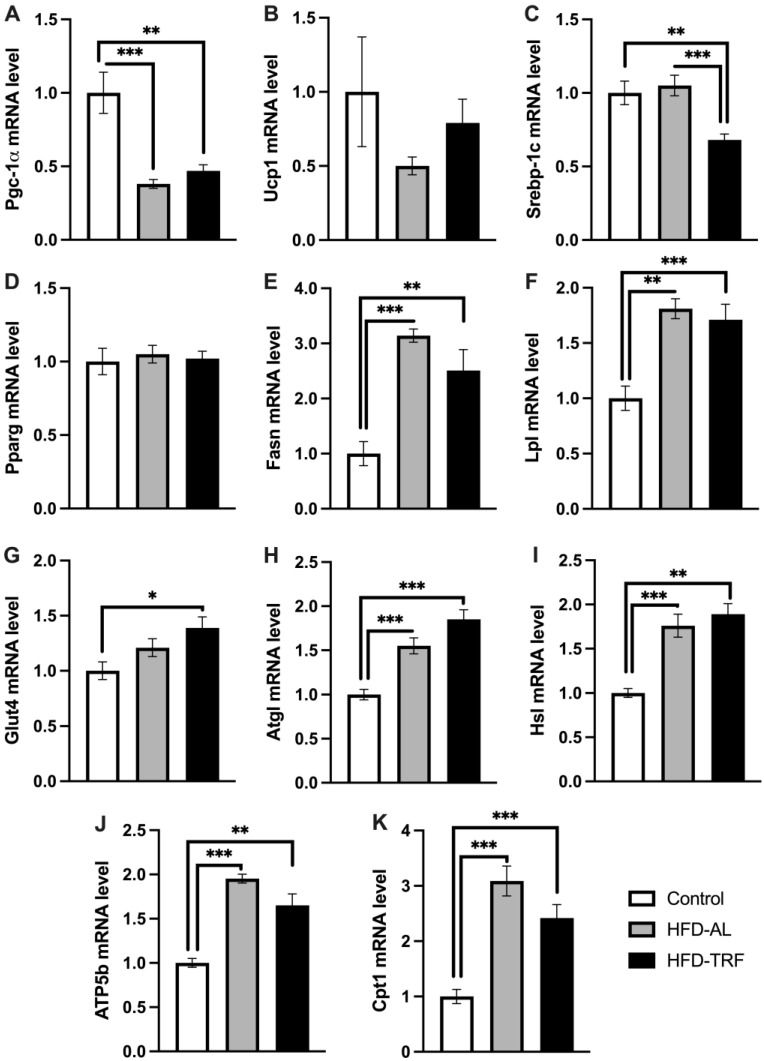
Lipolytic and adipogenic gene expression in inguinal white adipose tissue. (**A**–**K**) mRNA expression of genes involved in thermogenesis, adipogenesis/lipogenesis and lipolysis in inguinal white adipose tissue of control, HFD-AL and HFD-TRF mice. The data are represented as mean +/− SEM. * *p* < 0.05; ** *p* < 0.01; *** *p* < 0.001.

To determine if there was a white adipose tissue depot difference in the metabolic effect of TRF, the metabolic gene expression in inguinal white adipose tissue was also examined. Unlike epididymal adipose tissue, the expression of adipogenic genes did not follow a consistent trend within the inguinal adipose tissue in HFD-fed obese mice in response to TRF. *Srebp-1c* gene expression was significantly decreased by TRF compared to both HFD and control group while *Pparg* was not altered by either diet group (Figure 5A,B). *Fasn* and *Lpl mRNA* expression levels were increased by both HFD and TRF compared to control (Figure 5C,D). The mRNA expression of *Glut4* was increased by TRF compared to the control, while HFD had no significant impact (Figure 5E). *Pgc-1α* gene expression was significantly decreased by HFD compared to the control, and TRF had no reversal effect on *Pgc-1α* expression (Figure 5F). Next, the study looked to compare lipolytic gene expression in the inguinal adipose tissue to see how it alters by TRF compared to the epididymal adipose tissue in HFD-fed obese mice. The mRNA expression levels of *Atgl* and *Hsl* genes were found to be increased significantly within the inguinal adipose tissue by HFD, which then increased slightly further with TRF but not significantly compared to the HFD group (Figure 5H,I). Two genes involved in mitochondrial and fatty acid oxidation, *Cpt1* and *Atp5b* were upregulated significantly by HFD compared to the control group (Figure 5I,J). Both genes were reduced by TRF compared to the HFD group. These results indicate that HFD has an opposite effect to what was found in the epididymal adipose tissue, i.e., increasing the expression of both lipolytic and lipogenic genes in inguinal adipose tissue. Therefore, TRF has a minimal impact on lipid metabolism within the inguinal adipose tissue compared to the epididymal adipose tissue.

### 3.6. TRF Increases Free Fatty Acids and Decreases β-Hydroxybutyrate Concentration within Serum

**Figure 6:**
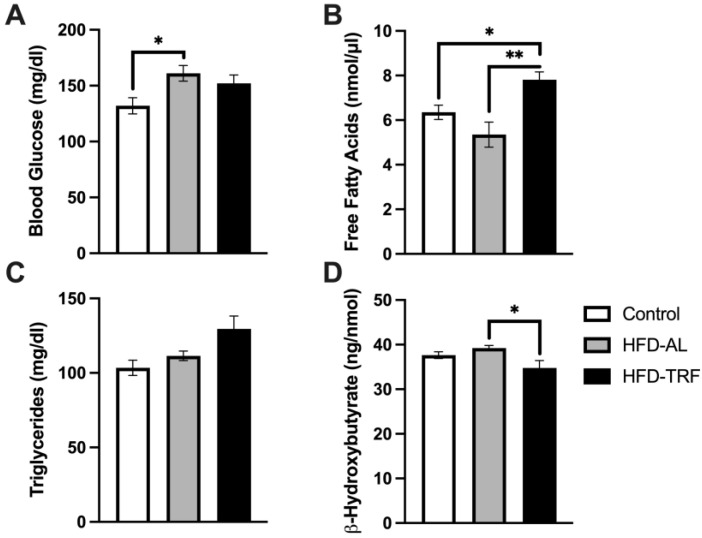
Serum glucose and lipid measurements. (**A**–**D**) Serum measurements of male mice with 16 h fast, *n* = 4–7/group. (**A**) Serum glucose (mg/dl), (**B**) free fatty acid (nmol/μL), (**C**) triglycerides (mg/dl), and β-hydroxybutyrate (ng/nmol). The data are represented as mean +/− SEM. * *p* < 0.05; ** *p* < 0.01.

To determine how TRF alters systemic glucose and lipid metabolism, serum was collected from three groups of mice after overnight fasting and analyzed for lipid levels. Blood glucose levels were analyzed via Glucometer prior to the mice being sacrificed and serum kits were used to determine the concentration of free fatty acids (FFA), triglycerides, and B-Hydroxybutyrate (Figure 6). The blood glucose level was significantly increased in the HFD group compared to control mice (Figure 6A). There was a slight decreasing trend found in the TRF mice compared with the HFD-AL group, but with no significance (Figure 6A). This decreasing trend leads to a partial restoration of blood glucose levels towards the control group. The TRF mice had significantly increased levels of serum FFA compared to both the control and HFD-AL groups (Figure 6B). There were no significant differences between groups for serum triglycerides, but the trend was increasing for the TRF group (Figure 6C). Lastly, the TRF group had decreased concentration of *β*-hydroxybutyrate compared to the HFD-AL with statistical significance (Figure 6D). Collectively, these results suggest that TRF partially improves blood glucose control, increases fatty acid release, and decreases serum ketone body levels in obese mice induced by a long-term (28-week) HFD feeding.

## 4. Discussion

In this study, we investigate the effect of TRF on body weight, adipose tissue morphology, and metabolic gene expression in brown, inguinal, and epididymal adipose tissues. The 10-week TRF regimen began after 18 weeks of HFD feeding in all mice minus the control group. The average weight of the control mice stayed consistent through-out the study with a slight increase (~1 g) towards the end of the TRF regimen. HFD group had a decrease in body weight shown in week 2 of the study, which is likely due to not allowing the mice sufficient time to adapt to being moved between cages two times per day prior to starting the TRF regimen. There was a significant difference found in the reduced body weight of TRF mice, which slowly increased back towards the initial starting weight between week 2–8 and stayed consistent the last two weeks of the TRF regimen, staying below the starting weight. Previous studies have shown TRF does reduce the percentage of weight gain in mice, however, most studies begin TRF while simultaneously starting the HFD [22,23,24]. The results in this study indicate that TRF is also an approach for effective weight loss when mice have already developed diet-induced obesity. Food intake data, which was collected by calculating the difference in food daily, showed no significant difference between the HFD and TRF groups (Figure 1B). Caloric density between the normal chow feed and high-fat feed were different which is likely why both HFD and TRF groups were significantly different from the control. When normalizing tissue weight to bodyweight, there was an increase in both inguinal and epididymal percentage for the TRF group compared to the HFD. This increase is not typically seen in previous studies [24,25,26]. One reason for this could be due to a longer HFD feeding duration (28 weeks) than the rest of the studies looked at, which is supported by previous studies [21,27]. For instance, Epi-WAT was found to be increased by short-term HFD but decreased by long-term HFD feeding which could be due to impaired adipogenesis. In this study, we also show that 28 weeks of HFD reduced the weight of epididymal adipose tissue. Although the mechanism for this phenomenon is not completely known, TRF seems to be able to prevent this decrease likely through restoring adipogenesis.

Since it is of importance to understand how TRF changes adipose tissue mass and morphology, fat cell sizing analysis was conducted, which is the first time this has been done in a TRF study, to the best of our knowledge. The results showed an increase in the average diameter of fat cells within the TRF group compared to the HFD for both the inguinal and epididymal adipose tissue. An increase in fat cell size or hypertrophic adipocytes is traditionally considered to correlate with system insulin resistance and metabolic dysregulation within humans [12,13,14]. Adipose tissue expands through two different mechanisms: hypertrophy and hyperplasia of adipocytes. Adipocyte hypertrophy through increasing the size of existing adipocytes is a common feature of dysfunctional adipose tissue occurring in obesity, which is caused by the impairment of adipogenesis. Conversely, adipocyte hyperplasia through increasing the formation of new adipocytes with normal function (adipogenesis) is associated with healthy adipose tissue expansion, as it can help store or sequester detrimental fatty acids in adipocytes, thereby reducing ectopic fat accumulation and improving metabolic health. The beneficial role of healthy adipose tissue expansion through adipogenesis or hyperplastic expansion of both subcutaneous and visceral adipose depots has been well documented [28,29,30,31]. For instance, increasing subcutaneous fat mass via inducing adipogenesis is considered as a beneficial mechanism for the anti-diabetic effect of thiazolidinediones, a class of *p*eroxisome proliferator activated receptor gamma (*Pparg*) agonists in insulin resistance and type 2 diabetes [32,33,34,35]. In a recent study, loss of mural cell *Pparg* could lead to pathologic visceral WAT expansion induced by HFD feeding. On a contrary, overexpression of *Pparg* in platelet-derived growth factor (*Pdgfrβ*) positive (adipogenic) precursors increases their adipogenic capacity leading to healthy visceral WAT expansion and improvements of glucose homeostasis in HFD-induced obese mice [36]. To get a better understanding of how TRF-caused expansion of inguinal and epididymal adipose depots was occurring, we further separated the data into size populations. The HFD group had an increased amount of smaller fat cells (preadipocytes) than the TRF group, while the TRF group had an increased amount of larger fat cells (fully differentiated adipocytes) and a decreased amount of smaller fat cells within both Ing-WAT and Epi-WAT, suggesting TRF affects (or expands) both depots similarly through increasing adipogenesis (the number of fully differentiated adipocytes). This suggests that the expansion of adipose tissue by TRF has a beneficial impact on metabolic improvement in obesity. *Cidea* expression, which is a lipid droplet-associated protein, has been reported to play a positive role in adipose tissue expandability and ameliorating the metabolic profile during diet-induced obesity [21]. We show that the expression of *Cidea* gene within brown adipocytes where it is highly expressed in mice was found to be significantly decreased by HFD and restored by TRF. The restored expression of *Cidea* implies that TRF promotes healthy expansion of adipose tissue, which may help preserve insulin sensitivity on HFD as reported in previous studies [37].

Next, we looked at how TRF changes both brown adipose tissue morphology and metabolism. TRF was able to reverse the HFD-induced whitening of brown adipose tissue, as seen in the histology of BAT. This is consistent with previous TRF studies conducted in mice [21,23]. This could be explained by the increase seen in *Atgl* gene expression within the BAT, contributing to fat mobilization, decreasing the lipid droplet size. However, genes involved in thermogenesis such as *Ucp1* and *Pgc-1α,* were not significantly impacted by TRF. *Pgc-1α* is a transcription factor that controls thermogenic genes, such as the expression of *Ucp1* [38]. *Ucp1* is known to mediate brown-adipocyte-specific non-shivering thermogenesis, which modifies whole body metabolism and susceptibility to weight gain [39]. Therefore, thermogenic properties are downregulated by HFD within the brown adipose tissue and TRF does not appear to be able to restore these changes. Similarly, in inguinal adipose tissue HFD significantly down-regulates the expression of both *Pgc-1α* and *Ucp1* genes, but TRF fails to restore their expression. Moreover, the mitochondrial biogenesis gene *Tfam* was increased significantly by TRF compared to HFD but *Errα,* and *Pgc-1α* did not show any change between the groups within BAT. Looking at mitochondrial and fatty acid oxidation genes (*Cpt1* and *Atp5b*), an increase was seen in both genes within the HFD group. *Cpt1* is known to facilitate fatty acids into the mitochondria for beta oxidation. However, a decrease in *Cpt1* and *Atp5b* gene expression was seen in the TRF group compared to the HFD group within the brown adipose tissue and inguinal white adipose tissue, suggesting the beta-oxidation of fatty acids is reduced in BAT in the TRF group compared to the HFD group. It has been reported that brown adipose tissue thermogenesis and fatty acid oxidation is diurnally regulated in humans [40]. Previous investigations have demonstrated that thermogenic genes are changed by TRF in a *rhythmic* manner; TRF upregulates *Ucp1* in the dark cycle but has no effect in the light cycle [23]. It is likely that no effect by TRF observed in our study could be due to the time point when the experiment was ended, as mice were sacrificed in the light cycle after a 16 h fast.

In addition to UCP1-dependent thermogenesis, several ATP-consuming futile cycles are known to play a role in energy dissipation, including mitochondrial ADP/ATP carrier (AAC)-mediated proton leak, SERCA2b-mediated calcium cycling, creatine-dependent ADP/ATP substrate cycling, and lipid cycling (lipogenesis/TAG synthesis and lipolysis) [41]. However, it is unknown whether the futile cycle mechanism is involved in the TRF regulation of weight loss. Interestingly, we found that HFD significantly reduces the expression of genes involved in both lipogenesis/adipogenesis (*Pparg, Srebp1c, Fasn, Lpl, Dgat*, and *Glut4*) and lipolysis (*Atgl* and *Hsl*), whereas TRF is able to restore the expression of these genes within the epididymal white adipose tissue. These results indicate that TRF restores the HFD-induced changes, increasing them back towards the control level, for the expression of adipogenic and lipolytic genes in Epi-WAT. These results collectively imply lipid cycling (an ATP-dependent futile cycle consisting of an anabolic segment and catabolic segment), i.e., lipogenesis and lipolysis, is decreased by HFD, whereas TRF can restore the lipid cycling in epididymal adipose tissue [41].

However, unlike the Epi-WAT, the inguinal white adipose tissue did not follow a consistent trend in adipogenic and lipolytic gene expression. Therefore, our results show HFD increases lipolytic genes (*Atgl* and *Hsl*) and lipogenic genes (*Fasn* and *Lpl*) in inguinal adipose tissue, which TRF had a minimal impact. The minimal effect of TRF on the lipid futile cycling could also be explained by the oscillatory regulation of lipid metabolism in inguinal adipose tissue as discussed above. In a previous study, it was seen within the Epi-WAT during HFD feeding that adipogenesis is initiated earlier, whereas subcutaneous fat can undergo hypertrophy for longer and maintain lower rates of adipogenesis even after two months [42]. Since we see a change in lipid cycling within the Epi-WAT sooner than the Ing-WAT in response to HFD, this could be an alternative explanation as to why there is a difference in TRF effect on lipid cycling and adipogenesis between the two white adipose tissues.

TRF appears to partially improve blood glucose control through increasing glucose utilization, based on the decrease in blood glucose levels presented compared to HFD. TRF increased serum free fatty acid levels which was expected due to the increased expression of the lipolytic genes, *Atgl* and *Hsl*, leading to an increase in the hydrolysis of triglycerides from adipose tissue. This could also be due to the increased fasting time between meals, which led to the increased expression of lipolytic genes by TRF to use fatty acids as an energy source. It is important to note, the lipolytic genes were only significantly increased within the epididymal tissue by TRF compared to HFD and not within the inguinal tissue. Lastly, TRF decreased serum ketone body levels which is likely due to enhanced ketone body utilization by the TRF group since they could have adapted and become more efficient under the fasted state during the 10 weeks of TRF regimen.

Potential limitations within the study include not letting the mice adapt to being switched between cages, which may have led to disruption of bodyweight at the beginning of the study between the three groups. Additionally, it is much easier to impose TRF on mice than it is with humans, and this should be considered when comparing our results with other studies and when considering implementing TRF.

## 5. Conclusions

We have demonstrated that TRF is effective for weight loss in HFD-induced obese mice and reduces weight gain under the long-term HFD feeding. TRF can restore the impairment of adipogenesis by HFD and increases lipid storage capacity in white adipose tissues. This shows obesity causes issues with the function of expansion within adipocytes, but TRF is able to increase and restore the expansion of adipocytes, therefore increasing the functional amount. We also found that TRF has a depot-dependent effect on lipid metabolism and restoes ATP-consuming futile cycle of lipogenesis and lipolysis that is impaired by HFD in epididymal adipose tissue. For the development of TRF-based dietary and lifestyle interventions, further investigations are needed to help understand the detailed mechanisms for the depot-dependent effect of TRF and its metabolic benefits.

## Data Availability

The authors confirm that all relevant data are included in the article and materials are available on request.

## Supplementary Materials

The following supporting information can be downloaded at: https://www.mdpi.com/article/10.3390/nu15010238/s1, Table S1: List of primers for RT-PCR analysis.
