# Supplementary material for: Depot-Dependent Impact of Time-Restricted Feeding on Adipose Tissue Metabolism in High Fat Diet-Induced Obese Male Mice
Source: Nutrients. 2023 Jan 3;15(1):238. doi: 10.3390/nu15010238 (PMC9823673; doi:10.3390/nu15010238)
Supplement: Supplementary file 1 [file nutrients-15-00238-s001.zip › nutrients-2101468-supplementary.pdf]

Supplemental Table S1

Gene name	Forward primer	Reverse primer
Ucp1	TAATGACTGGAGGTGTGGCAGTGT	TGTTGACAAGCTTTCTGTGGTGGC
Tfam	CACTGGGAAACCACAGCATACAG	GGACATCTGAGGAAAAGCCTTGC
Errα	CCAGACAGCAGCCTCAAAAAC	GATAGGGACCGAACACAGATCCT
Pgc-1α	ACCGTAAATCTGCGGGATGATGGA	AGTCAGTTTCGTTCGACCTGCGTA
Scd1	CACTGAATGCGAGGGTTGGTTGTT	TCCTTTCAGCAGCACTGTACCACT
Elov15	GGTGGCTGTTCTTCCAGATT	CCCTTCAGGTGGTCTTTCC
Cpt1	CCTCCCTGGGCATGATTG	ACGCCACTCACGATGTTCTTC
Atp5b	GCAAGGCAGGGACAGCAGA	CCCAAGGTCTCAGGACCAACA
Cidea	TGCTCTTCTGTATCGCCCAGT	GCCGTGTTAAGGAATCTGCTG
COXIV	ATGTCACGATGCTGTCTGCC	GTGCCCCTGTTCATCTCGGC
Atgl	TGTGGCCTCATTCCTCCTAC	TCGTGGATGTTGGTGGAGCT
Hsl	AGGTGGGAATCTCTGCATCACTGT	TGTCCCTGAATAGGCACTGACACA
Pparg	CAAGAATACCAAAGTGCGATCAA	GAGCAGGGTCTTTTCAGAATAATAAG
Lpl	TGAGAAAGGGCTCTGCCTGA	GGGCATCTGAGAGCGAGTCTT
Srebp-1c	CTTTCCTGGCTTGTCCTTTGGGA	GCTGGAAGGCAAAGGAACAACTGA
Glut4	GTAACTTCATTGTCGGCATGG	AGCTGAGATCTGGTCAAACG
Dgat	CTCTGCCACAGCATTGAGAC	TGCTACGACGAGTTCTTGAG
Fasn	CTGGACTCGCTCATGGGTG	CATTTCCTGAAGTTTCCGCAG
Tbp	ACCCTTCACCAATGACTCCTATG	TGACTGGAGCAAATCGCTTGG
